# Supplementary material for: Are carbon emissions trading and green financial instruments synergistic? -Comprehensive quantitative research based on content analysis
Source: PLoS One. 2024 Mar 7;19(3):e0298601. doi: 10.1371/journal.pone.0298601 (PMC10919601; doi:10.1371/journal.pone.0298601)
Supplement: S1 Data — (ZIP) [file pone.0298601.s001.zip › Data/地级市Z_F工作报告.zip!/║╙▒▒/╒┼╝╥┐┌╩╨/└┤╫╘╣1⁄2╓┌║┼í╛┬φ┐╦╩2╛▌═°í┐_2018.docx]

各位代表：

现在，我代表张家口市人民政府向大会报告工作，请予审议，并请市政协委员和列席会议的同志提出意见。

一、2017年工作回顾

2017年，在习近平新时代中国特色社会主义思想指导下，我们坚决贯彻落实习近平总书记视察张家口重要讲话和重要指示精神，按照省委、省政府和市委的总体部署，自觉践行新发展理念，统筹推进抓改革、促发展、惠民生、保稳定各项工作，经济社会各项事业实现了平稳有序发展。全市生产总值完成1555.6亿元、增长6.8%，全部财政收入完成256.56亿元、增长7.1%，一般公共预算收入完成135.8亿元、下降4.2%，固定资产投资完成1638.7亿元、增长0.5%，社会消费品零售总额完成752.5亿元、增长10.2%，实际利用外资40965万美元、完成省下达任务的102.4%，城乡居民人均可支配收入分别达到28512元和10293元、分别增长9.4%和11.4%，万元生产总值能耗下降6.5%。

一是冬奥会筹办步入新阶段。认真贯彻总书记“四个办奥”“五个着力”重要指示，落实省委、省政府“四个调整”要求，严格按计划推进各项筹办工作。编制完成了赛区规划设计方案并通过冬奥领导小组审定。列入2017年建设计划的越野滑雪场、冬季两项中心等38个项目全部开工，京张高铁、崇礼铁路主线工程已完成过半，延崇高速全面开工。大力培养冰雪人才，张家口学院组建冰雪学院，河北北方学院开设滑雪课程，我市青少年运动员5人入选国家队，在全国性比赛中获得4金5银2铜的好成绩。资助2.6万名青少年参加滑雪运动。举办自由式滑雪世界杯等19项国际国内赛事，组织了丰富多彩的冰雪活动，冰雪热持续升温，我市荣获“中国十佳冰雪旅游城市”。着力发展冰雪经济，高新区、宣化区2个冰雪产业园建设进展顺利，法国MND雪道缆车项目开始建设，与美国布鲁姆、瑞典万众之星等8家冰雪运动装备生产企业签订了落户协议，与国外8家机构建立了委托招商关系。

二是脱贫攻坚取得新成效。精准开展建档立卡“回头看”，全市贫困人口确定为404432人，贫困发生率13.02%。认真落实“五个一批”，积极推进脱贫攻坚和生态建设、新型城镇化、绿色产业发展“四方联动”扶贫开发新机制，产业扶贫覆盖326695名贫困人口，其中村级光伏扶贫电站并网27.6万千瓦，可带动34495户贫困户年均增收3000元。易地扶贫搬迁开工建设65个安置小区，安置人员20525人。改造农村危房19066户。解决162个贫困村、132698名贫困人口安

全饮水问题。为10264名贫困人口提供了生态护林员公益岗位。对284250人次学生实施了教育资助，为9567名建档立卡贫困生落实了“三免一助”政策。建立起贫困人口多重医疗保障体系。华大基因在张北县开展适龄妇女和新生儿免费基因筛查，探索了一条健康扶贫新路子。2017年全市有229个贫困村、116195名贫困人口脱贫出列。坚决贯彻习近平总书记和中央领导同志的重要批示精神及省委、省政府决定，深入开展了扶贫领域腐败和作风问题专项治理。

三是生态建设再上新台阶。推进蓝天、绿地、碧水、净土行动，完成造林绿化222万亩，林木绿化率由39%提高到43%。新增及改善节水灌溉面积44万亩，地下水超采基本得到控制。建立了四级河长制，地表水国省控监测断面水质优良比例达到87.5%。尚义察汗淖尔国家湿地公园试点通过验收，蔚县壶流河、阳原和涿鹿桑干河获批国家湿地公园试点，官厅水库国家湿地公园加快建设，尚义大青山国家森林公园通过评审。全市退出矿山90处，拆除燃煤锅炉1645台，整治“散乱污”企业431家，淘汰老旧车4292辆。空气质量综合指数4.18，PM2.5年均浓度31微克/立方米，空气质量达标天数286天，连续四年保持京津冀地区最好水平。首都水源涵养功能区和生态环境支撑区规划通过专家评审。京津冀协同办上报的支持“两区”建设的建议报告得到中央和京津冀协同发展领导小组同意，省委、省政府正在制定支持意见，“两区”建设全面提速。

四是转型升级实现新提升。新旧动能转换加快，呈现出了“稳、进、好、新”的态势。“稳”的态势在持续。在大力调整结构、去产能的情况下，全市生产总值、规模以上工业增加值增速与上年基本持平。居民消费价格指数上涨1.9%，保持了稳定。“进”的力度在加大。压减粗钢216万吨、煤炭283万吨。完成工业技改投资118.1亿元。规上工业产业结构持续优化，装备制造业、可再生能源业占比达到34.8%，超过煤炭、钢铁业23.5个百分点;高新技术产业同比增长11%，占比达到29%，同比提高3.9个百分点;战略性新兴产业同比增长19.9%，占比达到22.2%。服务业同比增长11%，对经济贡献率达到72%。全域旅游发展加快，成功举办了首届全市旅发大会和首届延怀河谷葡萄文化节，全年接待游客6259.8万人次，总收入696.5亿元，分别增长20.5%和34.1%。现代农业持续提质增效，产业化经营率提高0.5个百分点，达到64.5%。“好”的成效在显现。全年税收收入同比增长14.6%，金融、高端装备制造、可再生能源产业税收分别增长11.6%、19.4%和67.6%，财政收入质量大幅提高。“新”的动能在积蓄。一批重大产业项目实施或投产，沃尔沃发动机制造公司入选第一批国家绿色工厂，生产发动机13.6万台。

吉利沃尔沃领克汽车张家口工厂落成投产，10家汽车零部件企业落户园区，亿华通氢燃料电池发动机下线;阿里庙滩等4个数据中心项目和怀来秦淮大数据产业基地建设加快;“北方硅谷”(上海张江分园)主营业务收入和税收分别增长379.1%和306.9%，增速居全省经济开发区第1位和第2位;张北云计算产业基地创建为国家新型工业化产业示范基地;汽车制造、大数据、高新技术产业集群开始形成。“大创园(高创园)+孵化器+加速器+产业园”双创生态链初步形成，新增省级以上科技创新平台9家，其中国家级2家;科技型中小企业和高新技术企业分别增加350家和33家，分别增长30%和66%。

五是协同发展取得新进展。借助冬奥会，积极推进与北京的合作。京张“8对8”对口帮扶深入开展，实施帮扶项目68个，投入资金5.7亿元。京张沿线高铁新城规划基本编制完成。怀来航空航天产业园火星着陆试验场等部分项目开建，经开区金刚石切割线项目建成投产，北汽福田氢动力汽车、中科院雷达遥感综合试验场等一批重大项目加快推进。教育医疗合作面扩大，市级新增3家京张合作医院，24家公立医院与北京30家医院开展合作。中公教育国际学校、阿里巴巴培训学院张家口分院成立。与北京方面签订合作协议30项，总投资722.5亿元。与深圳、上海、浙江等地企业开展了多项合作，特别是深圳华大基因、建筑科学研究院等企业，已经在我市农业、健康、特色小镇等领域展开规划建设。

六是可再生能源示范区建设实现新突破。规模化开发稳步推进，新增装机112.7万千瓦，总装机容量达到1171.2万千瓦，并网1093.4万千瓦，占全部电力装机的71%。中科院太阳能储热、100MW压缩空气储能系统等12个示范项目取得积极进展。电网建设加快，“三站四线”工程全面投运，世界首个具备虚拟同步机功能的新能源电站投入运行，全球规模最大的柔性直流电网试验示范工程获得国家核准，多个国家级“互联网+”、微电网示范项目获批实施。大力推进可再生能源应用，完成电供暖413万平方米。金风科技风电装备基地、晶澳太阳能组件宣化和康保基地开工建设，英源高效光伏组件、龙驰科技锂电池项目完成注册。国内首创的“四方机制”可再生能源电力交易平台正式运行，3个月挂牌交易电量6730万千瓦时，为可再生能源综合应用奠定了良好基础。

七是城乡面貌呈现新气象。城市总体规划修编报告已上报住建部。实施投资3000万元以上城建重点项目282项，完成投资320亿元。沙岭子西站如期投入运行，奥体中心开工建设。棚户区改造完成5.76万套。集中建设限价房1.2万套，其中中心城区5587套，回购、建设人才安置房385套。集中开展“双违”和“一区四边”整治工作，拆除违建570万平方米。解决房地产开发遗留问题354个，办理房产证4.82万个。中心城区五马路、容辰大街等12条道路实现通车。我市入选全国“城市双修”、老旧小区改造和土地利用总体规划修编试点城市;荣获首批国家食品安全示范城市、河北省文明城市、河北省卫生城市。涿鹿县成功创建国家园林县城。开工建设27个省级中心村示范点，71个村达到省级精品村标准。交通建设顺利推进，大张、张呼铁路工程进入扫尾，京新、京蔚高速快速推进，新改建农村公路626.5公里、维修160公里。全市常住人口城镇化率达到55.9%。侯卫东官场笔记

八是重点领域改革迈出新步伐。持续推进“放管服”改革，市县两级行政审批局挂牌运行。编制完成权力清单、负面清单15张。全面推行“多证合一”“双随机一公开”，新登记市场主体46269户，增长21.8%。行政执法“三项制度”改革取得阶段性成果。积极拓展投融资改革，全年新增新三板等挂牌企业16家。争取债券资金62.89亿元，到位国储林贷款13.2亿元、国开行和农发行棚改贷款111.8亿元。新组建3家市属一类国有企业，市属国有企业注册4支基金。开发区四项重点改革取得积极进展，经开区率先完成薪酬制度改革。农村土地经营权确权颁证工作基本完成，土地流转面积381.4万亩，流转率达到37.03%。医药卫生体制综合改革持续深化，公立医院综合改革、药品采购“两票制”全面推开。理顺物业、公厕管理体制，实现管理重心下移。农业水价和水资源税改革走在全省前列。

九是社会事业得到新加强。民生领域支出381.8亿元，占公共预算支出的81.05%，比去年增加48.5亿元。积极扩大就业，去产能企业职工分流安置4345人，新增城镇就业7.17万人，农村劳动力转移就业3.65万人，落实退役军人政策、再就业9615人。加快教育事业发展，全市有8所学校完工或投入使用;55所普惠制幼儿园和438个农村中小学“全面改薄”工程开工建设。积极改善医疗卫生服务，市第四医院新院区投入使用，县区5所二级医院在建或投入使用，4家三级医院开展医联体建设。完善社会保障体系，企业退休人员人均月增养老金149.52元;完成城乡居民医保整合，开通跨省异地就医实时结算定点医院20家;城乡低保年保障标准分别提高到6000元和3400元;新建农村幸福院100所，农村互助养老人数达到2327人。文化事业不断发展，我市剧作家参与创作的河北梆子剧《李保国》荣获全国“五个一工程”奖，地方戏剧《泥土情》等5部作品获得省“五个一工程”奖，举办了一系列大型文化节会活动，组织文化惠民演出活动400多场，元中都遗址获批河北首个国家考古遗址公园。平安张家口建设扎实推进，建成了市县两级综治中心，深入开展“雷霆2017”行动、“打黑除恶”专项行动，打掉涉黑恶痞霸团伙143个，阳原县荣获全国社会治安综合治理工作“长安杯”。人大代表建议和政协委员提案办结率100%。国防动员、双拥共建和优抚安置工作进一步加强，民族宗教、新闻出版、外事侨务、人民防空、史志档案、气象地震、地理信息、援疆援藏、妇女儿童、老龄、残疾人等事业都有了新发展。

二、2018年目标任务和主要工作

各位代表!过去一年的成绩来之不易，这是以习近平同志为核心的党中央坚强领导的结果，是贯彻省委、省政府和市委决策部署，全市人民齐心协力、共同奋斗的结果。我代表市政府，向全市人民，向人大代表、政协委员，向各民主党派、工商联、无党派人士和人民团体，向驻张解放军、武警官兵和政法干警，向中省直驻张单位，致以诚挚的感谢和崇高的敬意!

各位代表，在肯定成绩的同时，我们也认识到存在的问题和困难。一是对扶贫项目、资金监管不严格，监督执纪不到位，扶贫领域发生了腐败和作风突出问题，侵害了群众利益，损害了党和政府的形象。说明我们在思想、作风方面还有明显差距。二是实体经济经营困难，融资难、融资贵、融资慢问题普遍存在。三是民生社会领域还有不少短板，广大人民群众还有不少操心事、烦心事，对上学看病、道路拥堵、停车困难、物业管理等方面还有不少意见。安全生产、社会稳定等领域还存在风险隐患。四是农村人居环境普遍较差，基础设施和公共服务提升任务十分艰巨。五是政务环境、营商环境需要进一步改善。有的政府部门工作不扎实、效率不高，少数干部懒政怠政、推诿扯皮，影响着发展环境和地方形象。此外，由于土地占补平衡收入锐减、去产能、产业转型升级不够快等多种原因，一般公共预算收入、固定资产投资、工业增加值没有完成年初计划。我们必须强化问题导向，持续精准用力，下大功夫解决。

2018年是全面贯彻党的十九大精神的开局之年，是改革开放40周年，是决胜全面建成小康社会、实施“十三五”规划承上启下的关键一年。做好政府工作，要全面贯彻党的十九大精神，以习近平新时代中国特色社会主义思想为统领，深入贯彻落实习近平总书记视察张家口重要指示，牢固树立新发展理念，加强党对一切工作的领导，围绕中央、省系列重要部署和市委十一届三次全会确定的“七个着力”基本思路，按照高质量发展的根本要求，坚持“创新竞进、协同融合、改革开放、转型升级、提质增效、改善民生、优化环境”，以冬奥会筹办和脱贫攻坚为统揽，以推进供给侧结构性改革为主线，以改革创新为动力，立足“建设好首都水源涵养功能区和生态环境支撑区”，加快构建绿色生态体系、绿色城镇体系、绿色能源体系、绿色产业体系、绿色交通体系，努力打造体育之城、活力之城、康养之城、文明之城、富强之城。

主要目标是：生产总值增长7%，规模以上工业增加值增长5%，固定资产投资增长15%，社会消费品零售总额增长10%，一般公共预算收入增长7%，实际利用外资增长5%，城乡居民人均可支配收入分别增长9%和11%，万元生产总值能耗下降3.8%。PM2.5年均浓度30微克/立方米，主要污染物减排完成省下达目标任务。居民消费价格指数涨幅控制在3%以内，城镇登记失业率控制在4.5%以内。

各位代表，中国特色社会主义进入新时代，我国社会主要矛盾已经转化为人民日益增长的美好生活需要和不平衡不充分的发展之间的矛盾。解决我国社会主要矛盾，必须推动高质量发展。我们要重视量的发展，但更要重视解决质的问题，在质的大幅提升中实现量的有效增长。要坚定不移贯彻新发展理念，坚定推进供给侧结构性改革，把推动高质量发展作为当前和今后一个时期确定发展思路、制定经济政策、抓好各项工作的根本要求，推动经济社会在实现高质量发展上不断取得新进展，更好满足人民在经济、政治、文化、社会、生态等方面日益增长的需求，更好推动人的全面发展、社会全面进步。

今年着重抓好以下工作：

(一)肩负重大政治责任，努力办好国家大事。筹办冬奥会、脱贫攻坚和京津冀协同发展是党中央的重大战略部署，是我们必须全力以赴办好的国家大事。

1.坚持科学有序、集约节约，高标准扎实推进冬奥会筹办工作。认真贯彻“四个办奥”理念，切实履行主办城市的主体责任，聚焦赛事核心，高标准推进各项筹办工作。同时，因势发展奥运经济，推进冰雪运动、冰雪产业在张家口落地生根。

高质量推进场馆和配套设施建设。进一步细化时间表、路线图、责任制，有序高效推进，确保将冬奥项目建成优质、生态、人文、廉洁的精品工程。已经开工的万龙到转枝莲道路、崇礼城区输水等43个项目，要保质量、抓进度，用好“一会三函”，尽快完善手续。抓紧各项准备，确保崇礼客运枢纽、张家口南综合枢纽等22个项目按期开工，奥运村5月份全面开工。强化冬奥项目资金全程监管和跟踪审计，做到保质保量、节俭廉洁。

全面开展各领域服务保障工作。全面启动人才培养、后勤保障、市场开发等各项工作。加快冰雪运动和相关人才培养，抓好张家口学院冰雪学院、北方学院和市职教中心相关专业建设，支持宣化二中建设国家体育总局青少年户外学训基地和全国首所体教结合的冬奥专业学校。加强与国外冬奥城市的交流合作，学习借鉴办赛经验。积极开展赛会志愿者和城市志愿者的招募培养和培训工作。完成住宿保障规划及实施方案，兼顾赛事需求和赛后利用，结合村庄撤并，在崇礼启动建设一批公寓式酒店和庄园式民宿。按照冬奥食品体系建设规划及实施方案，全面推进冬奥食品供应基地和供应链建设。加快冬残奥会无障碍环境建设。组织开展“全民迎冬奥、当好东道主”系列活动，编写迎冬奥培训简本教材，分类实施，进机关、进课堂、进社区、进企业、进农村。多形式广泛开办“奥运讲堂”，多形式广泛开展迎冬奥文明创建活动，让我们的家乡越来越有国际范儿，让和谐文明之花在张垣大地竞相开放。

加快推广冰雪运动。多形式建设冰雪运动设施，鼓励支持各县区建设滑冰、滑雪场地，开办培训机构，组建市冬季运动管理中心。组织承办好国际体育大赛，举办一批参与度高、影响力大、带动力强的群众冰雪运动赛事，叫响“大好河山·激情张家口”冰雪运动品牌，鼓励和支持更多青少年上冰雪，打造以崇礼为中心的国际知名冰雪运动和冰雪旅游胜地、国家体育休闲综合示范区、国家冰雪运动推广普及中心。

因势发展奥运经济。借力冬奥筹办加快改善城乡环境，完善城市功能，提升城市品质，提高城市美誉度和吸引力。通过冬奥筹办引进战略合作伙伴，加快发展信息技术、生态环保、商贸物流、人工智能、大数据、新材料、通用航空、食品加工等产业。结合京张体育文化旅游带建设，促进冰雪运动与旅游、科技、文化、会展、传媒等产业融合发展。加大招商引资力度，加快冰雪装备产业园建设，年内初具规模，为建设国家冰雪运动装备生产基地打下坚实基础。推进冰雪产业研究院和冰雪大数据平台建设，积极申报综合保税区。

启动2021年世锦赛筹办工作。推进组委会组建，建立高效工作机制。梳理世锦赛筹备任务清单，建立台账，扎实推进。科学安排特许经营计划、赞助计划和器材采购计划，注重选择对我市绿色发展有积极促进作用的企业和机构作为世锦赛合作伙伴，实现互利共赢、共同发展。

2.坚持集中资源、聚焦重点，坚决打好精准扶贫攻坚战。把脱贫攻坚作为头等大事和第一民生工程，突出目标导向和问题导向，统筹资源，精准发力，进一步加大扶贫力度，提升脱贫质量。

精准实施“五个一批”工程。聚焦深度贫困县，精细落实各项扶贫政策和“三个新增”要求，大力推进基础设施和基本公共服务双提升工程，改善发展条件，争取享受国家支持深度贫困地区的政策，利用好土地增减挂钩指标跨省交易政策。全面推进产业就业扶贫，实施职教扶贫工程，推广“互联网+扶贫”新模式，力争年内实现风电、光伏、特色产业、农牧业、服务业、加工业等优势产业联结贫困村、贫困户全覆盖。支持扶贫产业项目购买商业保险，确保扶贫资金安全，增强项目抗风险能力。今年完成60万千瓦风电、108万千瓦光伏电站建设和并网，与已建成的27万千瓦光伏电站，全部链接到贫困人口，带动13.2万贫困户稳定持续增收。通过土地流转、入股分红、劳动就业等方式，建立完善贫困户与龙头企业、平台企业、农民合作社的利益联结机制，实现稳定收益。加快推进易地搬迁扶贫，启动740个自然村包括24276名贫困人口在内的62311人搬迁，完成农村危房改造2.8万户。积极推进生态扶贫，以组建造林扶贫合作社、以工代赈等形式，组织有劳动能力的贫困人口参与生态建设，贫困人口护林员达到2.5万人。扎实做好教育、医疗扶贫，完善贫困家庭学生救助机制，实行县域内住院“先诊疗后付费”和“一站式”报销。坚决落实政策兜底扶贫，全面推广大病商业保险，编织四重保障兜底网。贫困县区普遍建立特困救助基金，贫困人口集中的地区要设到乡镇一级。提高扶贫大数据平台建设水平，用信息化手段实现上下联通、痕迹化管理，动态掌握贫困人口情况，推动各项帮扶措施的落实。力争年内崇礼、宣化、万全、涿鹿脱贫摘帽，157648名贫困人口达到脱贫出列标准。

全面推进“四方联动”扶贫开发新机制。积极借助北京对口帮扶我市13个贫困县区和中省直单位定点扶贫、省“五包一”资源优势，充分发挥驻村工作队作用，切实在落地扎根致富项目、引进企业和人才、改善基础设施等方面取得更多实效。大力支持县乡绿扶公司、扶农公司在脱贫攻坚、生态建设、城镇化、绿色产业发展“四方联动”中发挥好基础作用，把扶农公司打造成精准脱贫的战斗堡垒、绿色发展的商务平台，加快土地流转，谋划包装项目，广引市场主体，发展致富产业，广纳农民就业。把绿扶公司打造成贫困县区获得长期收益的资本平台，通过实施“金融+可再生能源”，积极参与、加快推进扶贫光伏和风电建设，为巩固脱贫成果提供财力补充。

严格落实脱贫攻坚各项责任。汲取教训，举一反三，全面加强监督执纪问责，持续开展扶贫领域腐败和作风问题专项治理。严格管好扶贫项目和资金，认真执行“谁拨款、谁监管”跟踪管理责任制，实行市直相关部门领导干部包县区捆绑考核制度。认真落实项目资金全程公开公示制度，自觉接受社会各方和广大村民监督。加强财政、审计、监察部门的全程监管监督监察。坚持发现问题立行立改，违法违纪立即追究，决不能再让少数害群之马抹黑广大干部群众的奋斗成就，扰乱张家口的大好局面。

3.坚持主动对接、全面融合，推动协同发展向深度和广度拓展。抢抓京张联合办奥的重大机遇，以承接北京非首都功能疏解为“牛鼻子”，加速与北京多方面对接融合，打造河北“一翼”，与北京的“两翼”共同形成保障首都、服务首都、共赢发展的新格局。

加快搭建协同平台。在高速、高铁、主要干路沿线、洋河新区和环京三县重要节点区域，高标准规划建设高铁新城、特色小镇、专业园区，打造协同发展、协同创新平台，大力推进与北京连接的交通项目建设，搞好商业、教育、医疗等相关配套服务，重点打造北京机构、企业、人才的事业新基地、生活新家园。加快怀来航天产业基地、张北云基地、桥东中关科技谷等平台项目建设，加快主城区高铁新城总部基地规划和项目可研，力争开工建设。加快京东大数据产业园、中国电信呼叫中心洽谈对接，力争年内开工。

大力引进优质教育医疗资源。努力争取国家教育部、卫计委和北京市的支持，在已有合作基础上，大力引进各级各类优质教育、医疗机构，采取合作、托管及建设医联体、分院、分园、分校等多种形式，实现更多合作项目实质性落地。推动华北电力大学科教基地、西城职教学校张家口基地、北理工怀来校区加快落地。大力推进洋河新区健康城、教育城建设，努力打造高端医疗教育资源集聚的龙头平台。努力拓展北京对口帮扶范围，与北京多层面、多领域建立更加紧密的联系，开展更广泛的合作，实现双赢发展。

积极推进特色小镇建设。充分发挥我市交通便捷、生态优良的比较优势，从供给侧着眼，坚持全域旅游、休闲康养和产业导入并重，深化协同发展规划，制定扶持政策，明晰合作模式，广泛寻求实力伙伴，谋划建设疗养康复、影视艺术、金融、体育、科技、文化等一批特色小镇，开辟协同发展的新领域。今年推动张北华大基因未来康养小镇、下花园金融家小镇、沽源丰源店国际假日小镇、怀来桑园葡萄小镇、塞北荣盛农旅小镇等20个以上特色小镇开工建设。已经开工的赤城海陀小镇要在产业导入、生态建设和脱贫攻坚方面发挥好先行示范作用。

(二)扎扎实实推进首都水源涵养功能区和生态环境支撑区建设，发展绿色经济，建设绿色城市。坚决贯彻落实习近平总书记视察张家口重要指示，加强生态建设，树立生态优先意识，建设好首都水源涵养功能区和生态环境支撑区，探索一条绿色发展、生态强市的路子。在积极向国家、省争取支持的同时，不等不靠、主动作为。

1.坚持综合治理，加快构建绿色生态体系。推进山水林田湖草气系统治理，坚决打好污染防治攻坚战，张开首都生态保护伞，促进绿色现代化发展示范区建设。

持续开展蓝天行动。大力开展工业企业全面达标排放行动，彻底整治“散乱污”企业，全面推进散煤和燃煤锅炉治理，加快以可再生能源为主的清洁能源替代，全年完成电供暖700万平方米以上，压减煤炭消费20万吨。严格煤尘面源精细化管控，开展车油路专项整治，年内淘汰老旧机动车5000辆。大力整治建筑工地扬尘和渣土车遗洒，发现违规问题严肃追究责任。加强秸秆综合利用处理，加快秸秆集中处理体系建设。严格环境执法监管，严厉打击环境违法违规行为。

持续开展绿地行动。创新机制，市场运作，多方筹资。鼓励支持项目化、公司化、林场化造林。年内完成植树造林600万亩，林木绿化率达到50%，构建乔灌草结合、多层次、立体化的复合植被系统。发展林业产业，扩大经济林比重，积极探索森林旅游休闲，开展森林草原认领，推动生态资产交易，开发森林多功能效益，促进可持续发展。强化造林责任制，对造林情况进行全面核查，确保造林成效。加强退耕还林、退耕还草工作，大力建设农业公园、森林公园和草原公园。坝上四县年内各创建一个示范草原公园和示范牧场。完成土地利用规划修编工作，启动生态保护红线区域勘界定标，加强湿地保护和自然保护区管理，加快矿山退出和生态修复，年内退出矿山111处，治理水土流失面积200平方公里。

持续开展净土行动。编制实施土壤污染治理与修复规划。加强农业面源污染防治，建立固体危险废物全过程监管制度。大力推进有机肥替代工程，大幅减少化肥使用量。实施畜禽粪污资源化利用整县(区)推进，年内粪污资源化利用率达到80%以上。禁止使用0.01毫米以下厚度地膜，完善全市农膜回收体系。加快垃圾分类回收处理和一体化处理体系建设，推进无害化处理、资源化利用。

持续开展碧水行动。制定实施全市水资源保护利用综合规划，全面落实河长制、推行湖长制，加强河流断面监测，年内地表水三类及以上水质比例达到50%以上。工业园区全部实现污水集中处理，促进中水回用。启动桑干河、清水河、洋河、永定河、东沙河等综合治理工程，积极推进退耕还湿、退灌还水，在坝上四县开展2万亩退水还旱、旱作雨养种植试点，在怀来实施3万亩湿地生态恢复治理项目。严控地下水开采，保护好“首都水塔”。

2.坚持城乡融合，加快构建绿色城镇体系。大力实施乡村振兴战略，全面推进城市更新，加快实施“五级两规一导则”，加强城乡规划统筹、合理布局。着力提升基础设施和公共服务功能，促进城乡融合发展。

大力实施乡村振兴战略。按照产业兴旺、生态宜居、乡风文明、治理有效、生活富裕的总要求，突出重点，抓住关键，优先向农村配置资金、要素和公共服务，促进农村全面进步、农业全面升级、农民全面发展。一是大力提升农业发展质量。对标冬奥食品体系标准，坚持质量兴农、绿色兴农，实施农业供给侧结构性改革三年行动计划，推进农业由增产导向向提质导向转变、规模型向质量型转变。发展质量农业，大力推行标准化生产，年内新建标准化基地30万亩，有机农业基地5万亩。大力实施龙头企业提质扩模带行业计划，支持龙头企业发挥示范带动作用。发展科技农业，强化科技推广，加强与首农集团、华大基因等战略伙伴合作，建立技术支撑体系，启动蔬菜种子基因库、农业研究院建设。发展节水农业，大力压缩耗水作物，推广高效旱作品种，新增高效节水灌溉面积10万亩。发展品牌农业，创建张北马铃薯、怀涿葡萄全国特色农产品优势区，做强燕麦、口蘑、杂粮、杏扁等特色农产品品牌。实施“互联网+现代农业”，大力转变农业生产、经营、流通方式，促进农村一二三产业融合发展。二是大力改善农村人居环境。优化村镇布局，因村实施乡村改造提升或撤并新建工程。开展农村人居环境整治三年行动，以“厕所革命”、垃圾污水处理、燃煤置换、“四好农村路”为重点，推动农村基础设施建设提档升级，加快改善农村生产生活条件，建设低碳宜居乡村。引导农民积极参与，探索多种改造建设模式，量身定制支持政策，积极引进市场主体，采取经济、实用、高效、可持续的运行模式，促进村容村貌快改善，农民生活品质快提升。上半年启动100个左右“四到位”示范村镇，带动全市农村全面展开。加强农村突出环境问题综合治理，持续搞好乡村绿化工程，建设秀美乡村。三是大力推动农民全面发展。加大农村新型经营主体培育力度，发展农村股份合作经济。大力发展休闲农业、乡村旅游、农村电商等新产业、新业态，多渠道促进村集体和农民增收。抓好新型职业农民培训，支持新型职业农民通过弹性学制参加中高等农业职业教育。支持职业院校利用教育培训资源，为农村培养一批农业职业经理人、经纪人、乡村工匠，进一步强化乡村振兴的人才支撑。鼓励社会各界投身乡村建设，带动农民整体素质提高。四是大力加强乡村治理。扎实做好村委会换届选举工作，把有责任心、有带富能力的人充实到村委会班子。推进村务公开，完善村规民约，加强村级民主监督和民主管理。加大农村普法力度，加强农村法治建设，推进法律援助进村、法律顾问进村，严厉打击村霸和宗族恶势力，推进平安乡镇、平安村庄建设。传承发展农村优秀传统文化，广泛开展农村移风易俗活动，加强农村思想道德文化建设，促进形成文明健康的生活方式。

大力推进城市更新。抓好新一轮城市总体规划修编实施。市县同时实施城市“双修”、基础设施改善、市容提升、老旧小区改造、棚户区改造、路网畅通六大攻坚行动，提高城市承载功能。常住人口城镇化率达到57%。对标雄安新区规划理念，高标准建设高铁新城和洋河新区，打造“未来之城”。启动詹天佑文化轴建设，加快张家口学院新校区建设。中心城区实施一批重要市政基础设施及配套工程，新建18条城市道路，打通城市道路节点11处，完成纬二桥主体结构工程，建成城市餐厨垃圾、医疗废弃物无害化处置设施。大力推行绿色建筑、装配式建筑，支持住宅产业化发展。上半年基本完成房地产开发遗留问题和“双违”整治。年内所有县城达到“洁净城市”标准。

大力加强城市管理。强化“以人为本”思想，理顺管理体制，深入推进城市管理综合执法体制改革，实施城市管理领域行政处罚权集中行使，搞好城管与相关部门的工作衔接，推动执法重心向基层延伸。全面推行“三定一挂”工作机制，定人员、定网格、定标准，与工作奖惩挂钩。严格城管责任体系，努力做到无缝隙、全天候精细管理。明确完善城市管理标准，重点解决好道路施工、广告牌匾、随意遛狗等方面和城乡结合部的管理顽疾，严格规范作业。大力开展全国文明城市、全国卫生城市创建活动，发动群众广泛参与，自觉纠正违规和不文明行为，促进市民素质和市容环境双提升。进一步优化交通标识标线、信号灯配时和隔离带设置，试行单行循环线路，加强智能交通系统建设，研究实施信号灯“绿波”工程，增加道路巡警配置，大力发展公共交通，综合施策，解决交通拥堵问题。出台中心城区停车场建设管理办法，多渠道、多形式建设停车场地，规范住宅小区地面和地下车位管理，打造智慧停车系统，有效缓解停车难。切实加强物业管理，支持组建辖区物业管理集团，大力解决老旧小区脏乱差、公共设施破损、侵占公建公共空间等问题。

3.坚持提质增效，加快构建绿色产业体系。围绕高质量发展的根本要求，聚焦壮大实体经济，持续推进“四大两新一高”产业发展，推动经济转型升级。

突出推进重点项目建设。加大精准招商力度，实行横向联合、纵向协作、捆绑式考核的招商新模式，制定完善产业招商目录，确定重点招商对象，引进一批科技含量高、投资规模大的好项目。对重点招商项目梳理汇总，实行台账管理，全程跟踪协调服务，提高项目落地速度和建设速度。加强开发区建设，落实《张家口市开发区招商引资若干优惠政策》。增加土地储备，创新土地供应方式，支持入园企业采取长期租赁、先租后让、弹性年期出让、土地年租制、共有建设用地使用权等用地方式，降低土地使用成本。抓好“创坝”二期、南山汽车城、海淀中关科技谷、高新区生命科学园、哈工大工业孵化器、宣钢高新技术产业园、怀来航空航天产业园等一批专业化园区建设，省级开发区主营业务收入增长15%以上，经济开发区创建为国家级开发区。实施吉利沃尔沃领克汽车、北汽福田氢燃料动力客车、中科院雷达遥感综合试验场等市级以上重点项目200项，当年完成投资500亿元左右。加强项目储备，市级储备100个亿元以上、县级储备10个亿元以上产业项目。

加快建设创新张家口。实施科技创新三年行动计划。大力支持创新平台建设，推动一批高新技术项目列入国家科技冬奥专项和可再生能源示范区科技专项，努力争取国家、省重要科技基础设施、实验室、技术成果转化平台布局张家口。推广使用科技创新券，支持企业引进科技人才、建立研发机构、购买创新服务。年内力争新增市级以上创新平台100家，其中省级以上10家。实施高新技术企业和科技型中小企业倍增计划，年内新增高新技术企业50家、科技型中小企业400家。大力推动“双创”，市县(区)两级因地制宜建设创客空间、创业工场、创新创业社区，年内新增孵化器3家、创客空间20家。大力实施人才工程，落实市委引进人才的各项政策，重点引进和培育一批有核心技术、有专利成果、有创业项目的高水平创新团队和创新人才，以及园区管理人才、教育培训人才。创新资本运作方式，组建张家口金融服务中心和担保集团，建立政府产业引导基金、市级融资担保基金运营体系。积极引进股权投资、风险投资、创业投资，加快构建面向产业发展的多元化、多层次、多渠道投融资体系。积极与证券公司等加强合作，实施上市企业培育计划，实现域内企业在“新三板”挂牌5家以上，力争“主板”上市1家以上。

打好转型升级硬仗。在“破”“立”“降”上下功夫，坚持市场化、法治化手段，倒逼“僵尸企业”全部出清。制定《支持工业经济加快发展的措施》，支持规上工业企业建立研发中心，提高工艺技术、产品研发和管理运营水平，增强市场竞争力。全面落实重点企业联系服务机制，推动土地、金融、技术等要素资源向实体经济聚集。新增规模以上工业企业50家以上。落实“降税减证提标”三项措施，力争全年降低企业成本44.7亿元以上。深入开展《中国制造2025》和“互联网+”行动，用新技术、新业态、新模式改造提升传统产业，推动制造业加快向高端、绿色、智能方向转变。年内实施50项重点工业项目，培育10个智能工厂和数字化车间。大力支持工业设计发展，开展工业设计大赛，打造“设计+”产业链。

促进新动能快速成长。落实战略性新兴产业发展三年行动计划，积极发展大数据与物联网、人工智能、高端装备、信息技术、生物技术等产业，加快发展汽车制造、大数据及服务器制造、新能源与智能电网、可再生能源装备制造、冰雪运动装备、军民融合装备等一批产业集群。扎实推进“大智移云”引领计划，以政务云、企业云为重点，抓好“1+N”朵云智慧城市建设，加快大数据从物理存储、信息采集向数据分析、产品研发应用拓展，推动大数据在各领域、各行业的广泛应用，带动上下游产业发展。大力培育数字经济、共享经济、现代供应链等新业态新模式。深化与涉军单位合作，注重发展航空航天、先进制造等军民融合产业，加快建设怀来军民融合产业示范园区。积极发展现代物流、检测检验、金融后台、呼叫中心等生产性服务业和健康养老、娱乐休闲、家政服务等生活性服务业。

以高质量供给扩大消费需求。实施消费促进、电子商务进农村进社区、商贸物流配送体系建设、实体商业转型升级四大专项行动。加快发展文化体育、康养休闲等服务性消费。以创建国家全域旅游示范区为载体，抓规划、定政策、定标准、扶主体、强服务，使旅游业在各县区蓬勃发展。加强草原天路管控、整治、开发，打造户外休闲旅游胜地。推动云顶和万龙滑雪场联合创建全国第一个以滑雪为主题的5A级景区，加快桑洋水道、张家口堡等优质旅游资源高水平开发，提升现有景区景点品质，高标准包装开发地方旅游产品，推动旅游业由观光游览向休闲体验转变，实现旅游业总收入增长20%以上。办好第二届全市旅游发展大会，力争张北、蔚县创建国家全域旅游示范区获得成功。

深入推进质量强市建设。落实《开展质量提升行动加快质量强市建设的实施意见》，推动标准、质量、品牌、信誉联动提升。强化标准引领，建设经济社会新型标准体系，支持智慧互通、亿华通、旗帜、巡天、雪川等龙头企业参与制定或修订国际、国家、行业标准，培育一批标准领跑企业。落实省质量提升十大专项行动和质量管理提升“双千双百”工程，鼓励我市企业争夺国家、省质量奖、工业大奖，培育大批“河北工匠”、“张家口工匠”。实施百项新产品开发、百项品牌培育“双百”工程。注重诚信体系建设，整合信用资源，促进信息共享，推进征信大数据建设，健全守法联合激励和失信联合惩戒机制，引导市场主体靠信誉赢市场、以信誉换效益。

4.坚持综合创新，加快构建绿色能源体系。围绕可再生能源示范区“三大创新”“四大工程”“五大功能区”建设，抓重点、补短板、强弱项，大力推进低碳奥运专区和低碳城市建设，普惠群众生活，助力绿色崛起。

统筹发储输，建设京津冀绿电供应基地。积极推进规模化和分布式电站建设，开工风电300万千瓦以上、光伏100万千瓦，完成1080个村级光伏扶贫电站建设任务，年内可再生能源装机达到1300万千瓦，并网达到1200万千瓦。搞好大容量储能，完成中科院跨季储热、100MW压缩空气储能示范项目，争取尚义抽水蓄能电站核准开工。推进智能化输送，加快建设±500千伏柔性直流工程，争取张北-雄安新区1000千伏特高压输电项目补充列入国家电力发展“十三五”规划。

多措并举，促进可再生能源综合应用消纳。争取有关方面支持，加强与国网的协调配合，依据规划调整，努力加快城乡电网建设和提升，完成电网投资82亿元。努力拓展“四方机制”，制定适当政策和实施方案，在大力推广可再生能源供暖的基础上，积极向以电替油、绿色产业和生活用电延伸。年内可再生能源消费占终端能源消费的比例争取达到23%以上。全面启动崇礼低碳奥运专区建设。实施《氢能示范城市发展规划》，建设氢能利用体系。

强化技术支撑，完善产业链条。深化与中科院、国际可再生能源署、华北电力大学、北京鉴衡中心等有关机构和科研单位的合作，联合组建可再生能源研究院，布局一批可再生能源技术创新示范项目，争取国家可再生能源实验室落户我市。举办首届“张家口·长城国际可再生能源大会”，增进国际交流，扩大城市影响力，聚集优质项目和资源。启动农业可再生能源循环利用示范区建设，加快可再生能源装备产业园建设，年内，金风科技、中环光伏组件等6个项目投产。

5.坚持公交优先，加快构建绿色交通体系。大力改善交通路网结构，优化出行方式，发展便捷高效、安全可靠、低碳环保的绿色交通。

畅通城乡路网。构建大交通、拉开大框架，打造立体综合交通体系。实现京新高速三期、二秦高速建成通车，加快建设京蔚高速西段，启动建设京新至京藏(鸡鸣驿至涿鹿)高速连接线。进一步完善县域路网规划，加强县与县、乡与乡之间互联互通，京尚高速张北至冀蒙段、张北至康保一级公路开工建设，开展崇礼至张北段高速前期工作。实施1200公里通村畅乡、交通产业扶贫项目，建设“四好农村路”。开工建设张家口南、崇礼、下花园等综合客运枢纽。争取主城区至张北磁浮公交开工建设。启动区域通航线路前期工作。

推动绿色出行。推进国家“公交都市”创建工作，加快建设“优先发展公共交通示范城市”。科学设置、调整公交线路，加密公交车次，延迟收车时间，试行大站快线，谋划建设“智轨”公交线路。加大市域客运公交化改造力度，建设便民公交体系。公交出行分担率达到22%。中心城区保留的154部燃油公交车全部淘汰，新增200部氢燃料电池公交车、100部纯电动公交车。大力推广新能源车替代燃油出租车和社会车辆，发展200部网约车，推广纯电动、插电混合动力小型客车6000辆以上。

适度超前谋划。加快绿色交通基础设施建设，中心城区新建一批充电站、加氢站，实现充电桩建设在干路和主要公共场所全覆盖，并积极向社区延伸。各县区绿色交通基础设施建设也要全面启动，为电动车加快推广打好基础。严格落实安全设施，年内望山制氢厂投产运行。建设城市慢行系统，支持单车、徒步出行，谋划建设清水河冰上马拉松休闲健。

(三)坚持以人民为中心，进一步发展社会事业。针对关系人民群众切身利益的问题精准施策，加大投入，尽力而为，补齐短板，提高保障和改善民生水平。

加快发展教育事业。加大学前教育投入力度，年内55所普惠制幼儿园主体工程完成60%以上。着力解决中小学课外负担重、“大班额”“择校热”等突出问题。调整优化中小学校布局，促进城乡义务教育一体化发展。实施高中阶段教育普及攻坚计划、中等职业教育质量提升工程和高等职业教育创新发展计划。推进市职教中心扩容提升。深化职称和薪酬制度改革，提高一线教师、艰苦地区教师福利待遇。加强师德教育，治理有偿家教，招引优秀学科教师，进一步提升教师队伍整体素质。

促进就业创业。实施高校毕业生、职业学校毕业生就业创业促进计划，做好去产能分流职工、就业困难人员等重点群体就业工作。大力推广“职业院校+企业+农民”培训模式，加强职业技能培训，积极组织劳动力转移，多渠道增加农民经营性、工资性收入。年内新增就业8万人。

增强医疗保障服务能力。深化“三医联动”和公立医院综合改革，推进医联体建设和分级诊疗，提高家庭医生实际签约率，扩大服务范围。推进基层医疗卫生机构标准化建设，建立便民医药配送体系，全部开通村卫生室医保刷卡系统。大力引进优质医疗资源，多途径吸引和培养医疗人才，切实提高医疗卫生综合服务能力。支持市重点医院组织“移动医院”，在贫困县开展巡回医疗。完善计划生育服务管理，拓展基因筛查服务范围。

健全社会保障体系。完善城镇职工基本养老保险和城乡居民基本养老制度，新增企业职工养老保险参保1.95万人。完善最低社会保障和社会救助制度，城乡居民低保标准分别提高到7200元和4000元。建立农村留守儿童、妇女、老年人关爱服务体系。鼓励社会资本兴办城乡养老机构，加快推进医养结合基地建设，大力推进农村互助幸福院建设。加快老龄事业和产业发展。落实好企业军转干部、军队退役人员、优抚对象等有关政策，对困难人员及时给予援助。关注失独家庭，多形式关爱帮助。

保障群众基本住房需求。落实“房子是用来住的，不是用来炒的”定位，建立多主体供给、多渠道保障、租购并举的住房制度。以低收入住房困难家庭为重点，加大公租房保障力度。推进46个城中村和棚户区改造项目，新开工2.6万套，实施老旧小区改善项目39个8332套。加快推进人才安居工程。实施差别化调控，努力保障引进人才和本地居民刚性住房需求。组建国有房屋租赁公司，大力发展规范的住房租赁市场。

推进文化体育强市建设。加快推进京张体育文化休闲带建设。实施文化惠民工程，加强文化产业示范园和示范基地建设，抓好文物保护利用和文化遗产保护传承。支持主流媒体深度融合发展。组织好“红红火火中国年”民俗文化活动。大境门景区重新开放，对堡子里景区进行建设提升。广泛开展全民健身活动，推进县级全民健身活动中心、乡村农民体育健身、城市社区15分钟健身圈工程建设，打造“全季·全体育”活动品牌，支持各类体育组织发展，打造“运动之城”。

加强和创新社会治理。推广“共同缔造”理念，加强基层社区建设，推动公共服务职能和社会治理重心向基层转移，使政策落实在一线、问题解决在一线，共创文明社区。有效防控安全生产风险，全面构建安全生产一张网责任体系。健全公共安全体系，提升防灾减灾救灾能力。提高食品安全综合治理能力，巩固扩大国家食品安全示范城市创建成果。推进企业监管信息共享平台建设，形成一处违法、处处受限的震慑效应。有效防控社会风险，积极推进“雪亮工程”建设，完善立体化、信息化社会治安防控体系，构建新型矛盾排查化解常态化机制。扎实开展“扫黑除恶”专项行动，严厉打击各种违规违法行为，维护良好社会秩序，切实增强人民群众安全感和幸福感。加强全民国防教育、国防动员和后备力量建设，大力支持驻张部队推进新时代强军事业，着力解决随军家属就业和子女入学问题，发挥市县乡村“两站两中心”作用，做好军队退役人员安置和服务工作。充分发挥工会、共青团、妇联等人民团体桥梁纽带作用。继续做好民族宗教、新闻出版广电、外事侨务、人民防空、史志档案、气象地震、地理信息、援藏援疆、妇女儿童、老龄、残疾人等方面工作。

(四)全面深化改革，激发绿色发展强大活力。按照中央的总体部署和顶层设计，认真落实省委、省政府和市委对改革的具体要求，强力推进重点领域改革。

深化“放管服”改革。以建设“网上政府”“身边政府”和“最多跑一次”为目标，构建市、县(区)、乡(镇、街道)、村(社区)四级政务服务平台，充实专职办事队伍。加快市县(区)之间、部门之间信息系统、数据资源互联共享，搭建全市统一的网上大厅和移动客户端，实现申请、受理、审核、发证、公示全部一网通办。继续整合行政事项，实现信息共享。对目前市级保留的135项行政审批事项逐项推敲，或精简、或下放、或暂停、或合并，简化程序、优化流程。统筹推进“多证合一”，积极开展“双创”“双服”，着力提升“双随机、一公开”监管规范化、标准化水平。每月召开一次企业家座谈会，听取意见，及时协调，切实帮助解决问题。

深化国有企业改革。进一步完善市属国有企业法人治理结构，建立现代企业制度，积极推进多层次混合所有制改革。以管资本为主推动国资监管职能转变，建立出资人监管权力清单和责任清单，实施国有资产经营收益预算全覆盖。出台市属企业国有资产交易监管办法和重大投资监管办法。推动国有企业横向联合、纵向整合和专业化重组，做优做强做大国有资本。积极引进战略合作伙伴，加快基础设施、公共服务和城镇化建设。

深化金融监管体制改革。进一步规范政府举债行为，稳妥处置债务存量，有效遏制增量，切实防范金融风险。完善地方金融监管体制，严格落实金融监管职能，完善市、县(区)两级地方金融监督管理机构。根据“属地管理”和“谁审批、谁监管、谁担责”的原则，落实地方金融归口监管。重点开展对投资(咨询)公司、互联网金融、房地产中介机构、开展信用互助的农民专业合作社等地方金融、类金融组织的风险排查，严厉打击非法金融活动。统筹推进农业农村、医药卫生、税收征管、开发区、招商体制机制等领域改革，充分释放改革红利。抓改革、促管理、强服务，打好防范化解重大风险攻坚战。

三、忠诚履职尽责，努力建设人民满意政府

新思想引领新时代，新时代呼唤新作为。我们必须认真学习、深刻领会、坚决贯彻党的十九大精神，自觉用习近平新时代中国特色社会主义思想武装头脑、指导实践、推动工作，讲学习、转作风、抓落实，努力建设法治政府、效能政府、廉洁政府。

要旗帜鲜明讲政治。自觉强化“四个意识”，坚持党对一切工作的领导，把维护以习近平同志为核心的党中央权威和集中统一领导作为最高政治原则和根本政治规矩。推进“两学一做”学习教育常态化制度化，扎实开展“不忘初心、牢记使命”主题教育，坚定理想信念，筑牢信仰之基。始终做到心明眼亮、蹄疾步稳。

要牢固树立新发展理念。坚决贯彻党中央“五位一体”总体布局和“四个全面”战略布局重大决策部署，把创新、协调、绿色、开放、共享的新发展理念贯穿到各项工作的方方面面，努力使创新成为第一动力、协调成为内生特点、绿色成为普遍形态、开放成为必由之路、共享成为根本目的，坚定推进供给侧结构性改革，推进质量变革、效率变革、动力变革，谋求高质量发展。

要全面推进依法行政。自觉接受人大法律监督、工作监督和政协民主监督，多渠道、多形式加强与民主党派、人民团体、工商联、无党派人士、社会各界的沟通联系，广泛听取意见、建议和批评。深入贯彻法治政府建设实施纲要，全面落实司法体制改革要求，加强专业化法律服务，把依法行政、依法办事的要求贯穿于政府决策、执行、落实的全过程。推行行政执法三项制度，搞好“两法衔接”，严格规范公正文明执法，推进综合执法改革。特别要加大关系人民群众切身利益的重点领域的执法力度，以及时有力有效的实际行动提高政府公信力。

要强化督查抓落实。严格落实中央八项规定和实施细则精神，扎实推进纠正“四风”和作风纪律专项整治，深入开展“十查十改”。坚决整治尽职不力、作风散漫、推诿扯皮、纪律松散等现象。坚决克服形式主义、官僚主义，祛除享乐主义、奢靡之风。发扬钉钉子精神，狠抓各项工作落实，进一步提高效率、增强执行力、强化落实力。建立任务清单、问题清单、责任清单、措施清单、结果清单，定人、定事、定时、定质，严格实行督办、督查、监察递进督促问责机制，常态化运用第三方对重点领域进行调查评估，及时纠正政府工作中存在的问题。

要严守纪律规矩。把纪律和规矩挺在前面，切实加强政府系统党风廉政建设和反腐败工作，严肃查处侵害群众利益、侵吞国家财产的腐败问题，有效组织、持续用力、大力整治发生在群众身边的“微腐败”，坚决清除“细菌”和“病毒”。践行“三严三实”，始终向廉洁自律的高标准看齐，做到心中有党、心中有民、心中有责、心中有戒，敬畏人民、敬畏法律，努力建设一支忠诚履职、务实勤政、廉洁高效的公务员队伍。

各位代表，新时代新征程已经开启。让我们更加紧密地团结在以习近平同志为核心的党中央周围，在省委、省政府和市委的坚强领导下，高举习近平新时代中国特色社会主义思想伟大旗帜，不忘初心、牢记使命，锐意改革、勇于担当，团结协作、埋头苦干，对标新时代，制定新标准，展现新作风，干出新业绩，为完成好“三大历史任务”、交好冬奥筹办和本地发展两份优异答卷努力奋斗!
